# Platform gentrification: The production of urban inequalities in the on-demand city

**DOI:** 10.12688/openreseurope.20362.2

**Published:** 2025-11-11

**Authors:** Jorge Sequera

**Affiliations:** 1 Universidad Nacional de Educación a Distancia; Departament Sociology III, Madrid, Madrid, 28018, Spain

**Keywords:** platform gentrification; urban inequalities; platform urbanism;

## Abstract

This article introduces and theorises the concept of *platform gentrification* as a structural mutation in the production of urban inequalities under platform capitalism. Departing from the classical four characteristics of gentrification—capital reinvestment, arrival of new higher-status groups, landscape transformation, and displacement—this paper reinterprets these dimensions through the lens of the on-demand city, where digital rent platforms (e.g. Airbnb®), social media platforms (e.g. Instagram®), ride-hailing services (e.g. Uber®), and coworking companies (e.g. WeWork®) mediate, valorise, and restructure urban life. Rather than adding a new typology to the gentrification debate, platform gentrification is proposed as a critical framework to understand how algorithmic mediation, digital economies, and data-driven infrastructures reshape real estate markets, urban aesthetics, residential dynamics, and modes of exclusion. The paper argues that platform infrastructures not only organise mobility, consumption, and visibility, but also anticipate and accelerate new forms of displacement, both physical and symbolic. This concept is developed here as an interpretative tool particularly relevant for highly digitised urban environments, where the mediation of everyday life through platforms has become an invisible infrastructure of urban change.

## Introduction

Gentrification has long been one of the most fertile and contested concepts for describing processes of urban transformation under capitalism. Since its initial formulation as the displacement of working-class populations from central neighbourhoods by higher-income groups ( Glass, 1964; Slater, 2011), and its subsequent interpretation as a global strategy of accumulation by dispossession ( Smith, 2002), the concept has expanded to address new urban realities.

In the 2010s, the exponential growth of Airbnb and short-term rentals sparked a vast body of research on tourism gentrification and urban touristification ( Sequera *et al.*, 2022), examining the interplay between global tourism, skilled young migrants, and the reconfiguration of historic urban spaces ( Cocola-Gant & López-Gay, 2020; Jover & Barrero-Rescalvo, 2024; Milano *et al.*, 2024; Sequera & Nofre, 2018; Sequera & Nofre, 2020)). At the same time, scholars introduced the concept of transnational gentrification to capture the arrival of affluent international migrants and the transformation of neighbourhoods through real estate reinvestment and lifestyle-driven urban change ( Sigler & Wachsmuth, 2015; Hayes & Zaban, 2020).

Unlike touristification, which highlights processes driven by tourism as the main force of urban transformation, platform gentrification refers to a broader socio-technical regime where digital platforms mediate various forms of valorisation and exclusion, even when tourism is not the primary driver. Building on recent debates on assetization ( Birch & Muniesa, 2020; Gil, 2024), this article proposes the notion of *platform assetization* to describe how digital platforms convert urban space, mobility, and visibility into datafied and tradable assets. Rather than a new linear stage, platform gentrification should be understood as a structural mutation in the logic of urban accumulation—one that can be conceptualised through the emerging framework of platform assetization. 

Today, however, we are witnessing a new phase. Beyond the question of who is doing the gentrifying, the focus shifts to the ways in which the process is organised: through digital platforms that mediate, capture, and valorise urban space. This is not merely a new wave of gentrification, but a new urban regime. Digital rent platforms (e.g. Airbnb®), coworking companies (e.g. WeWork®), social media platforms (e.g. Instagram® and TikTok®), ride-hailing services (e.g. Uber®), and review platforms (e.g. Yelp®) not only facilitate the arrival of capital and mobile users, but also actively produce the urban environment in algorithmic, aesthetic, and economic terms.

In this article, I introduce and theorise the concept of *platform gentrification*. This hypothesis, situated within recent debates on platform urbanism ( Barns, 2020; Sequera, 2024) and the financialisation of housing through digital platforms and infrastructures ( Gil, 2024; Sadowski, 2020), does not seek to replace the classical concept of gentrification, but to reformulate it from a contemporary perspective that recognises the role that digital plays in the production of spatial inequality. To do so, I return to the four foundational characteristics synthesised by Davidson and Lees (2005: 1187) — *“*(1) the reinvestment of capital; (2) the social upgrading of locale by incoming high-income groups; (3) landscape change; and (4) direct or indirect displacement of low-income groups —and adapt them to the new scenario of multi-platform cities.

These four dimensions allow us to observe how gentrification is being reconfigured under the logic of platforms: how real estate investment is redirected towards algorithmic profitability and automated asset management; how platforms attract new urban consumers, such as transnational platform and tech workers, digital nomads, expatriates and global students; how they transform the urban landscape through digital aesthetics, visual interfaces, and filters influencing digital visibility and reputation; and how they generate displacement processes that are not only residential but also semiotic, digital and anticipatory.

In this sense, I propose the concept of platform gentrification as a hypothesis for thinking about how digital platform logics reshape the very conditions of possibility for gentrification: mediating accumulation practices, desirable subjects, urban aesthetics and exclusionary dynamics. Accordingly, I propose an update of the four classical characteristics of gentrification formulated by Davidson and Lees (2005), adapting them to the contemporary context of platformised cities. The objective is not to find a conclusive definition but to open a field of debate—one that is epistemologically hybrid and conceptually challenging—around the role of digital platforms in the creation of increasingly unequal cities.

## Towards a conceptualisation of platform gentrification

The ongoing digitalisation of cities, within a new globalising entrepreneurial urbanism characteristic of “Silicon Valley culture”, is capitalising on a constant flow of technological capital, which includes the opening of offices, consulting hubs, and the settlement of tech workers in strategic areas of the metropolis. ( Zukin, 2021). The relationship between platform capitalism and social change in urban society is accelerating through new consumption patterns, as the five most powerful tech corporations in the world—search engines and advertising platforms (e.g. Google®), hardware and software providers (e.g. Apple® and Microsoft®), e-commerce platforms (e.g. Amazon®), and social media platforms (e.g. Facebook®)— alongside iconic digital platforms—food delivery services (e.g. Deliveroo®), ride-hailing services (e.g. Uber), and digital rent platforms (e.g. Airbnb)—materialise across the city and emerge as key actors in its transformation. Their dominance over specific labour, economic and social spheres is closely linked to a technologised urban ecosystem, which we might consider the great platformisation of urban space ( Barns, 2019; Casilli & Posada, 2019; Nieborg & Poell, 2018; Pollio, 2021; Van Dijck *et al.*, 2018).

Thus, platforms operate as “regulatory structures”, not merely facilitating but dictating the terms of interactions and shaping user behaviour towards algorithmic processing and the monetisation of user data ( Peck & Philips, 2020; Van Dijck *et al.*, 2018). The platformisation of urban life and the platform economy should not be seen as simply the digitisation of products, services and business processes, but as a convergence of disruptions across various digital models, intersected by more agile forms of financialisation, the resurgence of labour regimes once thought obsolete (such as Taylorism, Toyotism and even techno-feudal forms of servitude), and heightened process opacity (slow regulation and black-box algorithms).

Today, this new phase of accumulation over urban space responds primarily to two overlapping dynamics: the digital (platformisation and intermediation) and the physical (property and infrastructure). Both generate value production, in terms of economic capital and data capital. The resulting spatial expansion patterns and alterations to the urban fabric vary depending on the platform economy and the assets involved. For instance, the type of platform operating in different areas of the city (central versus peripheral zones) determines a subject’s relationship to property and their labour role within the platform economy. In other words, in a platform city it is more common to see workers from the urban peripheries serving as the “labourers” involved in delivery services, ride-hailing platforms, and e-commerce infrastructures, while a rentier class of property owners can convert housing into assets and sublet them via digital rent platforms. Törnberg (2022) refers to this phenomenon through the concept of “platform place-making”, where urban platforms mobilise users as “discursive investors”, designing narratives and social infrastructures to shape extended imaginaries of place. For Törnberg, it is not just about transforming markets or opening up spaces for faster transactions, but about reconstituting the “perceptual fabric of space and socio-spatial practices within the urban”.

To capture the diversity of platform-mediated urban dynamics, I propose an operational typology ( Sequera, 2024) of urban platforms, as follows:

(i)   Spatial platforms: utilise properties, land and locations to deliver services (e.g. short-term and mid-term rental platforms such as Airbnb® and Uniplaces®);

(ii)   Chronotopic platforms: leverage specific infrastructures and temporalities for service provision (e.g. ride-hailing and food delivery services such as Uber and Deliveroo);

(iii)   Landscape platforms: represent and reconfigure the city visually (e.g. social media and streaming platforms such as Instagram, TikTok® and Netflix®);

(iv)   Interactionist platforms: operate in the public sphere, replacing or complementing conventional urban social relations (e.g. dating and review platforms such as Tinder®, Grindr® and Yelp), or in the private sphere, offering services within domestic life (e.g. task outsourcing platforms such as TaskRabbit® and cleaning service apps).

Thus, beyond channelling a range of services, platforms also produce social relations, symbolic hierarchies, modes of visibility, and ways of inhabiting a place. From rental and mobility apps to booking, recommendation and reputation interfaces, the contemporary city is not just built but programmed, filtered, and monetised. More importantly, platforms do not act as passive entities. Through social media and mapping platforms (e.g. Instagram, TikTok, and Google Maps®), for example, certain neighbourhoods become objects of desire via homogenised visual narratives, where specialty coffee shops, coworking spaces, street art and “authentic experiences” are represented as signs of modernity and symbolic capital. These images do not simply follow real estate investment; they precede and orient it. They reproduce a symbolic economy of place that links mobility, consumption and exclusion ( Bronsvoort & Uitermark, 2022; Leszczynski & Kong, 2023). Thus, we observe the emergence of so-called speculative digital enclaves: spaces simultaneously produced by data flows, brand value, and transnational physical presence.

From this perspective, platforms are more than just technologies; they are urban infrastructures that reorder governance, value production and spatial organisation within cities ( Barns, 2020). They do not represent an additive phenomenon, but an internal transformation of urban capitalism, in which the city becomes an interface for data extraction, process optimisation, territorial segmentation, and the hierarchisation of bodies. Platforms produce maps, rankings and representations that codify which areas deserve investment, which practices should be promoted, and which subjects are deemed legitimate.

This mutation is already visible across multiple global cities, from Lisbon, Barcelona and Mexico City to Rome and New York. These locations are home to a proliferation of urban typologies: Airbnbised districts, co-living spaces, coworking hubs and nomad neighbourhoods. These configurations no longer respond to traditional residential logic or stable productive structures; rather, they emerge from algorithmic intermediation and digitally projected symbolic value. Platform cities capture rents, channel mobility, and produce exclusions without the need for visible evictions.

Faced with this transformation, I propose understanding platform gentrification not as yet another “type” of gentrification, but as a new socio-technical regime of urban inequality production and socio-spatial segregation. In this sense, we return to the four classical characteristics of gentrification—capital reinvestment, arrival of new higher-status groups, urban landscape transformation, and displacement ( Davidson & Lees, 2005)—to reinterpret them through the lens of platform urbanism. In what follows, I analyse how these historical dimensions are transformed by platform logics, configuring a new phase of global urban capitalism.

While this typology helps to disentangle the multiple roles of platforms, these categories are not watertight. Many platforms operate simultaneously across spatial, symbolic, and temporal domains, producing complex reconfigurations of urban imaginaries and mechanisms of assetization.

## The four characteristics of platform gentrification

Although recent works have analysed the role of digital platforms in processes of urban restructuring—such as the mediatisation of space, the algorithmic financialisation of housing, or the aestheticisation of gentrifiable neighbourhoods—, a systematic conceptual framework addressing platform gentrification as a structural phenomenon has yet to be formulated. In this sense, the concept of platform gentrification proposed here does not merely aim to add a new label to the theoretical repertoire, but rather to highlight a profound mutation in the logic of contemporary urban accumulation. By understanding platforms as urban infrastructures that produce value, visibility and exclusion, this article seeks to develop a theoretical and epistemological research proposal that examines the role of digital intermediation in the production of new urban inequalities. This new scenario does not replace previous dynamics; instead, it reorganises, accelerates and complicates them, articulating new forms of accumulation by dispossession mediated through digital devices, transnational income flows and algorithmic (in)visibilities. Here, we propose a reinterpretation of the four characteristics outlined by Davidson and Lees (2005), under the hypothesis of a new phase in the process: platform gentrification.

### 1. Capital reinvestment: housing value in the era of the digital landlord

This transformation exemplifies a broader process of *platform assetization*, in which housing, mobility and urban visibility are reorganised as data-driven assets. Through algorithmic valuation, platforms translate spatial practices into measurable, tradable units of value, reinforcing new rentier logics within urban capitalism. The first classical feature of gentrification was the revaluation of urban land through processes of capital reinvestment, such as building rehabilitation or infrastructure investment. Today, this logic has become digitalised and decentralised, giving rise to more fragmented, opaque and transnational forms of accumulation. In platform gentrification, capital reinvestment is no longer limited to physical renovation processes but occurs through technological infrastructures, digital platforms and new speculative investment vehicles. The expansion of digital landlords, proptech platforms, and digitised rental systems (e.g. Airbnb, WeWork, and Badi®) has enabled new forms of urban rent capture with unprecedented speed, opacity, and transnational scale. This capture operates not only through rental platforms but also via digital real estate marketplaces that facilitate property transactions and investment flows, reinforcing structural gentrification processes globally. Examples include Idealista® in Europe, Zillow® in the United States, and MercadoLibre Inmuebles® in Latin America. These platforms work synergistically with rental platforms, creating multifaceted pressures on urban housing markets and contributing to socio-spatial inequalities.

Digital rental platforms (e.g. Airbnb, Spotahome® and HousingAnywhere®) operate as rent infrastructures, allowing investment funds, multiowners or professional managers to mobilise real estate assets without the need for physical transformation. As Sadowski (2020) points out, we are witnessing the rise of the Internet of Landlords, where housing becomes a financial asset managed through interfaces, APIs and automated market data. This trend connects with what we have termed a polyplatformised environment ( Gil *et al.*, 2023), where accommodation supply dynamically adjusts across platforms via channel managers and predictive algorithms.

Real estate pricing and analytics tools (e.g. AirDNA®, Transparent®, Mashvisor®, AllTheRooms Analytics®, Beyond Pricing®, PriceLabs® and STR Insights®) have established themselves as crucial digital infrastructures for the operation of the global real estate market, providing advanced data analysis systems to investment funds, asset managers, tourist rental companies and public administrations. By producing, circulating and monetising information on profitability, occupancy and short-term rental dynamics, these platforms actively contribute to the reconfiguration of urban space under logics of financial value extraction, algorithmic planning and data-driven governance.

In this context, the case of mid-term rentals is paradigmatic. As Cocola-Gant and Malet Calvo (2023) show, mid-term rental platforms (e.g. Uniplaces® and Spotahome®) allow the reconfiguration of rental durations according to global market demand, creating a hybrid rental form that maximises profits while evading local regulations. In this scenario, cities are no longer merely residential spaces but interfaces for digitalised rent extraction ( Sadowski, 2020).

Furthermore, as Vo (2023) has demonstrated, the growth of tech employment in US cities has been associated with induced gentrification dynamics, although with complex and unequal outcomes regarding ethnic and racial displacement. In contexts such as San Francisco, the arrival of companies like Google and Amazon has not only brought visible transformations but also the financial transformation of urban land ( Maharawal, 2017).

Moreover, investment funds in tech startups have found in the city a space for valorisation without the need to physically build anything, where all they need to do to intervene in specific parts of the city is to finance platforms that promote more profitable residential, tourist or coworking uses. Fast (2024) has shown how coworking spaces become elitist enclaves, not only physically but symbolically, expressing a privileged digital territoriality. These forms of “intangible infrastructure” enable capitalist reinvestment without direct urban intervention, generating value through the connectivity, accessibility, aesthetics and digital reputation of places.

### 2. New social groups: mobile, connected and tech populations

In platform gentrification, the profile of the gentrifier has shifted from the classical professional, the local artist or the creative class—the new urban middle class described by Butler—towards the transnational remote worker, the global student, the digital nomad, the expat, the tech entrepreneur and the global freelancer. These are groups characterised by more than their income alone, but also by their possession of strong digital capital ( Sequera, 2025). These populations are functional to the global city because they temporarily occupy central urban spaces, consume localised services, and are easy for platform logics to capture.

As Brollo and Celata (2023) show, this type of floating population tends to be concentrated in well-connected neighbourhoods laden with symbolic value, generating tensions with stable and working-class populations. It does not necessarily cause displacement through direct conflict but rather produces it in social and economic terms, through its consumption capacity, digital visibility and alignment with dominant urban imaginaries. These temporary and flexible profiles are reinforced by the platforms themselves, which streamline the urban experience by minimising spatial frictions through digital mediation (e.g. review platforms such as Yelp), managing payments (e.g. digital payment systems such as Mastercard®, Google Wallet® and Bizum®), facilitating mobility and on-demand services (e.g. food delivery platforms, ride-hailing apps and digital mapping tools such as Deliveroo, Uber and Google Maps), and facilitating cultural integration through event discovery apps (e.g. Bandsintown®). These new residents no longer need to decode and understand the city themselves; platforms recode urban cultural meanings, making them easier to assimilate and understand (via urban influencers on Instagram, for example). This implies that new groups not only enter but also actively *produce* urban space according to their aesthetic, usage and temporal patterns, displacing traditional forms of inhabiting and appropriating a place ( Cocola-Gant & Gago, 2021).

Fast (2024), for example, shows that coworking spaces are organised not merely as workplaces but as aesthetic environments where a homogeneous community—white, professional, educated, English-speaking and territorially unattached—is promoted. This aspirational class represents what Leszczynski & Kong (2023) describe as the “technology-gentrifier class”, capable of translating their presence into visual, digital and cultural capital, becoming a central figure in dominant urban narratives.

Bronsvoort and Uitermark (2022) further explore how certain urban figures are amplified by platforms like Instagram, which act as filters influencing social visibility. The arrival of new groups implies not only a demographic change but also a reorganisation of the visible space: who appears, who represents the neighbourhood, and which images define it (consider the distorted realities produced by social media platforms such as Instagram and TikTok). This overrepresentation generates the symbolic exclusion of previous inhabitants and disables their agency over the narrative of place.

### 3. Urban landscape transformation and algorithmic aestheticisation

Gentrification, as we are now observing, no longer operates exclusively in the physical realm but also in the semiotic and digital domains. Social media and mapping platforms (e.g. Instagram, Google Maps and TikTok) construct new forms of urban visibility, prioritising certain aesthetics, uses and bodies over others. As Bronsvoort and Uitermark (2022) have shown, platforms like Instagram amplify the representation of gentrifiable spaces through homogenised images of authenticity, creativity and exclusive consumption, producing a new type of landscape aestheticised by algorithms and filters.

This process is closely related to the idea of “aesthetic serialization” ( Leszczynski & Kong, 2023), where neighbourhoods are transformed in accordance with global visual patterns: specialty coffee shops, coworking spaces, “Instagrammable” murals, and so forth. As a whole, this generates a homogenised visual urban narrative that imposes itself over pre-existing aesthetics. Platformisation restructures the fabric of everyday urban life, affecting how space is thought about, valued, remembered, perceived, produced and regulated, with parallel effects on its associated social relations. Aesthetic serialization can be empirically observed through the spread of globally standardised features – such as coffee shops, coworking hubs, and urban murals – which appear across cities from Barcelona to Mexico City, yet are framed through homogenised digital narratives. What discursive, geographic and organisational interactions structure this platform ecosystem? In the words of Sharon Zukin (2020), the “spatiality of technological production” describes an urban environment in which new sociocultural norms are promoted and recreated within the urban fabric: coworking spaces or tech offices that reflect the power of tech entrepreneurs and their relationship with controlled land use as well as economic development policies. This aestheticisation is far from innocuous: it redefines the meaning of neighbourhoods and precedes their material transformation, anticipating future rent extraction and legitimising public or private interventions.

Thus, platforms not only transform buildings or businesses, but reconfigure the urban landscape through logics of digital aestheticisation, global serialisation and algorithmic recomposition of the visible. Platforms are more than mere intermediaries: they are active producers of urban space; they are shadow urbanists. Törnberg and Uitermark (2022) have shown how neighbourhoods like Vidigal in Rio de Janeiro become globalised stages through their circulation on video-sharing and social media platforms (e.g. YouTube®, Airbnb and Instagram). This process, which the authors call “urban mediatisation”, produces aesthetic and touristic value that does not necessarily correspond to the material conditions of the place.

Through this platform-mediated aesthetic transformation, neighbourhoods not only change in form but also in meaning: they become brands, products and destinations. The urban experience becomes “Instagrammable”, and urban policies compete to adapt their neighbourhoods to the pastel filters that generate desire, belonging and investment.

### 4. Displacement: anticipatory, digital and semiotic

Finally, platform gentrification redefines the very concept of displacement. Following Marcuse (1985), displacement has traditionally been understood as either direct (by means of eviction, rent increases or forced renovation) or indirect (the result of deteriorating living conditions, loss of services, or erosion of social capital). However, the platformised framework introduces forms of digital, semiotic and anticipatory displacement that operate even in the absence of physical movement. In this new cycle of gentrification, displacement no longer operates solely as direct expulsion but unfolds as a multi-layered process. Building on what Marcuse (1985) expounded, we recognise direct and indirect displacements, but extend this understanding to include anticipatory, digital and semiotic displacement, produced through the mediatisation and codification of urban space.

First, real estate pricing and analytics tools (e.g. Airbnb’s Smart Pricing, Beyond Pricing® and PriceLabs®) reconfigure market prices through predictive algorithms and continuous comparison, generating oversized and artificially inflated markets. This can trigger displacement as a consequence of value recalibration, whereby residents anticipate that they will no longer be able to afford to live in a given place, and leave before formal eviction occurs ( Vo, 2023). It can also lead to indirect displacement through exclusion for those who would wish to access upgraded areas, crudely segmenting the real estate market and urban zones.

Second, as discussed, semiotic displacement operates when residents no longer see themselves reflected in the neighbourhood’s image, lose digital representation or experience a hostile reinterpretation of their presence.

As Maharawal (2017) describes, the massive influx of tech workers into San Francisco, along with the proliferation of platform services and corporate shuttle buses, produced a form of cultural dispossession: not only did prices rise and were residents displaced, but the very meaning of “living well” in the city was redefined. Public spaces, everyday practices and traditional modes of inhabiting were displaced by a model of urbanism based on technological efficiency, algorithmic exclusivity and aestheticised consumption.

Third, digital displacement occurs: those who are not present on the interface simply do not exist. Those who are not visible on Instagram, for example, lack brand value. Studies such as those conducted in Amsterdam ( Bronsvoort & Uitermark, 2022) show that while some businesses gained high digital visibility thanks to professionalised influencers and foodies, others disappeared in digital terms, and eventually ceased to exist within the neighbourhood’s commercial landscape. Moreover, the algorithmic logic of platforms introduces a form of silent displacement based on the absence of data: those who are not in the system do not appear on the map. This invisibilisation further concentrates resources within the most visible and profitable territories, perpetuating urban inequality under the guise of technological neutrality.

Thus, platform gentrification generates displacement conditions from the very beginning of the gentrification cycle: anticipating land value, attracting new users, reconfiguring aesthetic landscapes and limiting access to certain bodies and practices. Displacement becomes cumulative and transversal—often invisible, yet radically effective. In this type of displacement, there is no need for physical eviction: it is enough to alter the code, the narrative, the use and the aesthetic. The right to the city is lost when the urban environment becomes unreadable, inaccessible or uninhabitable for those who do not fit within the platform interface.

Ultimately, platform gentrification redefines the very concept of displacement. The platformised framework introduces digital, semiotic and anticipatory forms of displacement that operate even without physical removal. These forms of displacement do not replace classical ones, but rather reinforce them. In fact, as this hypothesis proposes, the three previous characteristics—capital reinvestment, arrival of new groups, and aesthetic transformation—already generate the conditions for displacement from the outset, rather than it occurring as a final stage. Platform gentrification accumulates forms of exclusion throughout the process: invisibilising, disincentivising, aestheticising and excluding before physically evicting.

Displacement under platform gentrification becomes cumulative – as anticipatory, semiotic, and digital exclusions pile up – and transversal, operating simultaneously across housing, labour, and cultural dimensions. Within the framework of platform assetization, displacement is both a symptom and a condition of asset-based value creation. 

## Conclusion: naming the mutation to broaden the research agenda

Platform capitalism in the city manifests itself as markets tending toward new mechanisms of digital enclosure. Unlike the classical forms of urban capitalism, these platforms rely on the city but do not need to possess, purchase or own it. Instead, they own its circulation, its movement, its coordination, and the dialogues occurring among services, products, intermediaries, and citizens as client-consumers.

In this text, I have sought to coin the concept of platform gentrification, not to name yet another type of gentrification as a new category within the taxonomic repertoire of critical urbanism, but rather with the explanatory ambition of identifying a systemic mutation in how urban space is produced, represented and governed under the conditions of digital capitalism. Platform gentrification should be understood as an interpretative tool particularly useful for analysing contexts in which urban life is profoundly mediated, valorised and reconfigured by digital infrastructures, algorithmic flows and platforms of everyday intermediation. This mutation does not erase previous forms of gentrification but reorganises, accelerates and amplifies them through technical infrastructures that escape traditional scales of urban analysis.

As I have argued, platform gentrification must be understood as a process inherent to the platformisation of the city, where value extraction is articulated through data flows, algorithms, visibilities and transnational mobilities. Within this framework, urban land is not only economically revalorised but also symbolically and functionally redefined through logics of algorithmic codification, digital mediation and serialised aestheticisation.

The four classical characteristics of the gentrification process—capital reinvestment, arrival of new groups, urban landscape transformation, and displacement—do not disappear but are transformed into a continuous, anticipatory and decentralised cycle. There is no longer a clear linear sequence or distinct “wave”, but rather a distributed network of operations acting simultaneously across technological, financial, aesthetic and institutional fronts. The effects are no longer merely residential: they are also digital, semiotic and territorial.

Finally, platform gentrification coincides with a radical reorganisation of urban labour. While digital nomads, global students and freelance professionals are attracted by institutional incentives—visas, tax exemptions, urban branding strategies—the everyday reproduction of the city depends on a fragmented and precarious working class, comprising couriers, cleaners, delivery riders and other urban service workers mediated by platforms. This duality reproduces not only material but also symbolic inequalities in relation to visibility, recognition, access to rights, and the appropriation of space. Platforms do not merely reflect these hierarchies; they operationalise them through interfaces, filtering who appears, who has access and who counts. In the 21st century, reclaiming the right to the city also means contesting the right to visibility, to representation and to digital urban existence—in addition to defending the biographies that resist “offline living”.

This article advances a theoretical proposal and a research agenda aimed at understanding how states, far from passive, actively promote or regulate platform urbanism—often in ways that reinforce rather than mitigate platform gentrification. Ultimately, platform gentrification should be understood as one manifestation of a wider process of *platform assetization*—the conversion of urban life into a set of datafied and tradable assets. This perspective expands the lexicon of gentrification, revealing how platforms transform not only space but also value itself. In this sense, I have advanced *platform assetization* as a conceptual framework for analysing how platform capitalism restructures urban life through the assetization of housing, mobility, and visibility.

## Ethics and consent

Ethical approval and consent were not required for this study, as it did not involve human participants, personal data collection, or any form of empirical data gathering.

## Data Availability

No data are associated with this article.
