# Supplementary material for: Platform gentrification: The production of urban inequalities in the on-demand city
Source: Open Res Eur. 2025 Nov 11;5:147. Originally published 2025 May 28. [Version 2] doi: 10.12688/openreseurope.20362.2 (PMC12640495; doi:10.12688/openreseurope.20362.2)
Supplement: Peer review history

# Reviewer report: Reviewer response for version 2

**DOI:** 10.21956/openreseurope.23483.r63898

**Published:** 2025-11-22

**Authors:** Pascale Joassart-Marcelli

**Affiliations:** 1 San Diego State University, San Diego, CA, USA

I’m satisfied with the revisions. They strengthen the paper and make an already strong piece even more compelling.

Is the argument persuasive and supported by appropriate evidence?

Yes

Is the work clearly and cogently presented?

Yes

Is the topic of the essay discussed accurately in the context of the current literature?

Yes

Does the essay contribute to the cultural, historical, social understanding of the field?

Yes

Reviewer Expertise:

Urban Geography, Gentrification, Food, Tourism, Urban Aesthetics, Platform Urbanism

I confirm that I have read this submission and believe that I have an appropriate level of expertise to confirm that it is of an acceptable scientific standard.

# Reviewer report: Reviewer response for version 2

**DOI:** 10.21956/openreseurope.23483.r63899

**Published:** 2025-11-14

**Authors:** Luis Lozano-Paredes

**Affiliations:** 1 Transdisciplinary School, University of Technology Sydney,, Sydney, NSW, Australia

I thank the author for the revisions and thoughtful engagement with reviewer feedback. Below is a summary of improvements and a final assessment:

**Substantive Improvements**

 The revised version demonstrates significant theoretical advancement:

**1. Platform Assetization Framework** The introduction of *platform assetization* as an organising concept represents a significant contribution. This framework effectively situates platform gentrification within broader financialization processes and provides robust conceptual scaffolding that connects to current debates in economic geography and critical urban studies.

**2. Conceptual Clarity** The distinction between platform gentrification, touristification, and transnational gentrification is now clearly articulated. The author successfully establishes the specific analytical purchase of platform gentrification as a socio-technical regime rather than merely another gentrification typology.

**3. Geographical Scope**/ **Contextual diversity**. Mexico City appears consistently throughout the analysis, MercadoLibre Inmuebles is cited as a Latin American platform infrastructure, and Rio de Janeiro's Vidigal neighbourhood is discussed through the work of Törnberg and Uitermark. The discussion of platform workers building digital capital "collectively through informal learning and adaptation" gestures toward the creative appropriation dynamics I advocated for in my original review.

**4. Research Agenda** The conclusion successfully positions this work as both a theoretical contribution and a research agenda, including a discussion of state and corporate roles in platform-led transformations and methodological directions for future empirical work.

**Reconsidering Genre and Scope**

 I initially critiqued the paper for lacking empirical case studies. However, the author explicitly frames this as a "hypothesis," "interpretative tool," and "theoretical proposal". This genre is appropriate and indeed necessary for advancing conceptual frameworks. Foundational gentrification theory papers, including Davidson and Lees (2005), which this work reinterprets, were similarly conceptual rather than empirically exhaustive.

 The paper effectively cites empirical work by others to illustrate concepts, which is appropriate for establishing a new analytical framework. The author's goal is to "open a field of debate" and provide tools for future research, and the revised version accomplishes both.

**Productive Future Directions**

- Deeper engagement with platform cooperativism as alternative models
- More detailed analysis of community resistance and subversion strategies
- Comparative empirical studies demonstrating how platform gentrification operates differently across regulatory and cultural contexts

These represent opportunities for scholars to build on this framework.

**Final Assessment**

 This revised manuscript makes a significant theoretical contribution to platform urbanism and gentrification studies. The concept of platform gentrification, linked to platform assetization, provides valuable analytical tools for understanding contemporary urban transformation. The framework will stimulate meaningful scholarly conversations and empirical investigations.

 The paper succeeds in its stated aims: proposing a new interpretative framework, connecting platform processes to classical gentrification characteristics, and establishing a research agenda for future work.

Is the argument persuasive and supported by appropriate evidence?

Partly

Is the work clearly and cogently presented?

Yes

Is the topic of the essay discussed accurately in the context of the current literature?

Partly

Does the essay contribute to the cultural, historical, social understanding of the field?

Yes

Reviewer Expertise:

Design Research, Platform Studies, Science and Technology Studies (STS), Technology Governance, Urban Studies

I confirm that I have read this submission and believe that I have an appropriate level of expertise to confirm that it is of an acceptable scientific standard.

# Reviewer report: Reviewer response for version 1

**DOI:** 10.21956/openreseurope.22038.r54925

**Published:** 2025-07-21

**Authors:** Pascale Joassart-Marcelli

**Affiliations:** 1 San Diego State University, San Diego, CA, USA

This is an excellent article that introduces the concept of *platform gentrification* – a process which scholars have begun investigating and theorizing without naming and systematically defining it. Jorge Sequera provides a very organized overview of the relationship between the platformization of cities and gentrification, citing relevant literature. As such, it will be a useful frame of reference for scholars (including graduate students) embarking on research into gentrification and social exclusion in highly digitized urban environments.

 I appreciate that Sequera is not attempting to invent a new theory or typology of gentrification but instead productively reinterprets established tenets of gentrification in light of these new urban dynamics in which urban life is increasingly mediated by data-driven infrastructure and algorithms. He does so by revisiting the four classic dimensions of gentrification as defined by Davidson and Lees (2005), including new influx of capital, demographic shifts, landscape transformation, and displacement of longer-term residents.  This is an effective approach.

 There are a few points I wish the author would clarify or elaborate. Early in the article, there seems to be some ambiguity regarding the material and discursive reconstitutions of space caused by the platformization of urban economies. Sequera, discussing imaginaries of place and perceptual fabric of space, hints at the fact that much of this urban transformation happens semiotically and discursively. The relationship between material and discursive transformations and their significance in the current context, however, is not clearly articulated. The tension between cultural and political-economic explanations of gentrification have animated debates among urban scholars for decades. The discursive nature of gentrification is not new, but perhaps urban platforms amplify the significance of these dynamics relative to material changes.

 The typology of platforms presented on page 4 feels rigid and underutilized, since it is not used in subsequent parts of the article. For example, “landscape platforms” are defined as social media and streaming platforms that represent and reconfigure the city visually. However, platforms categorized here as spatial, chronotopic, and interactionist may also contribute to visual and symbolic redefinitions of urban neighborhoods. For instance, in my recent research Joassart-Marcelli 2025 (Ref 1) and Joassart-Marcelli and Bosco 2024 (Ref 2), platform-enabled services such as on-demand tourist experiences, restaurant reservations, or restaurant recommendations also shape neighborhood imaginaries and aesthetics, with impacts on residents’ everyday life.

 Throughout the article, there is the underlying assumption that platforms are altering the *phases* of gentrification and making the process less linear, but Sequera does not unpack the implications. Are “phases” equivalent to “stages”? Are we entering a new phase or are phases no longer relevant to understand contemporary gentrification dynamics? At the very bottom of page 4, the author writes: “we propose a reinterpretation of the four characteristics outlined by Davidson and Lees (2005) (Ref 3), under the *hypothesis of a new phase* in the process: platform gentrification.” He also describes the latter as “a new socio-technical regime.” Unfortunately, previous phases of gentrification are never discussed and the literature on this topic is not cited (for example Hackworth and Smith 2001 (Ref 4), Lees, Slater, and Wyly 2013 (Ref 5), Aalbers 2019 (Ref 6), Lees and Rozena 2025 (Ref 7) who have identified up to 5 stages). How does platform gentrification compare to what some scholars have described as fifth wave gentrification? How can a new phase be identified if previous phases have not been described?

 On page 7, the author concludes that “there is no longer a clear linear sequence or distinct ‘wave’, but rather a distributed network of operations acting simultaneously across technological, financial, aesthetic and institutional fronts,” but this idea was not flushed out in the analysis. Numerous gentrifications scholars have rejected linear notions of stages or waves, arguing for a more dynamics and multi-directional approach well before digital platforms transformed urban dynamics for example see Rose 1984 (Ref 8), Kerstein 1990 (Ref 9). I don’t think a complete review of theoretical work on different phases of gentrification is necessary, but a brief nod to past research and some clarification about how this current phase differs from previous ones would be helpful. The term mutation, which is also used in the article, might be more appropriate as it does not imply linearity.

 This new phase of gentrification presumably relates to the idea that “displacement becomes cumulative and transversal” (see page 7). This important statement is underdeveloped and could be expanded. How is displacement “cumulative” (or more cumulative than under previous urban regimes) and what exactly is meant by “transversal”? Sequera further hypothesizes that displacement occurs throughout the process of gentrification rather than being “a final stage” following economic, social, and aesthetic changes. I do not think many critical scholars would disagree with that statement. In fact, I would venture that most have understood displacement as a complex, cumulative, multifaceted, and dynamic process before the emergence of digital platforms. Yet, the platformization of cities make these dynamics more visible and provide an opportunity to reframe them.

 As I noted above, Sequera seems to suggest (without saying it outright) that, under platform gentrification, the discursive reconstitution of urban space is supplanting its material transformation. While acknowledging direct and indirect residential displacement, on page 7, he argues that “the platformised framework introduces digital, semiotic and anticipatory forms of displacement that operate even without physical removal.” The use of “introduces” is somewhat misleading. These forms of displacement are not entirely new—scholars have long discussed symbolic, psychological, and affective displacement—but their research might have been marginalized. Digital platforms do not create these processes, but they do intensify, operationalize, and render them more visible and systematic. In this respect, the article succeeds in foregrounding how platforms make visible a broader spectrum of dispossession than is often captured in mainstream gentrification studies.

 Finally, the article would benefit from greater engagement with the role of the state – a topic Sequera has addressed in earlier publications (see Janoschka and Sequera 2016 (Ref 10). Platform gentrification is not driven by platforms alone. To what extent does the state promote, regulate, or remain complicit in platform-led gentrification? Has its role been transformed—if not rendered irrelevant—by the rise of transnational digital capital? This is a crucial political question that deserves at least some discussion. Similarly, the question of power could be explored more deeply: while the article mentions digital nomads, students, and tech entrepreneurs, the platforms that mediate urban life are produced and governed by more powerful economic and political actors. Can the aesthetization of urban life and the transformation of real estate markets that platforms enable occur without input from the state and corporations set to benefit from continued capital accumulation by dispossession?

Is the argument persuasive and supported by appropriate evidence?

Yes

Is the work clearly and cogently presented?

Yes

Is the topic of the essay discussed accurately in the context of the current literature?

Yes

Does the essay contribute to the cultural, historical, social understanding of the field?

Yes

Reviewer Expertise:

Urban Geography, Gentrification, Food, Tourism, Urban Aesthetics, Platform Cities

I confirm that I have read this submission and believe that I have an appropriate level of expertise to confirm that it is of an acceptable scientific standard, however I have significant reservations, as outlined above.

## References

[1] : Eating like a local: Digital foodscapes, touristification, and gentrification in Paris’s Peer-to-Peer economy. *Geoforum* .2025;162: 10.1016/j.geoforum.2025.104287 10.1016/j.geoforum.2025.104287

[2] : ‘BEST FOR FOODIES ’: Food, Digital Media and Planetary Gentrification. *International Journal of Urban and Regional Research* .2024;48(1) : 10.1111/1468-2427.13212 74-93 10.1111/1468-2427.13212

[3] : New-Build ‘Gentrification’ and London's Riverside Renaissance. *Environment and Planning A: Economy and Space* .2005;37(7) : 10.1068/a3739 1165-1190 10.1068/a3739

[4] : The changing state of gentrification. *Tijdschrift voor Economische en Sociale Geografie* .2001;92(4) : 10.1111/1467-9663.00172 464-477 10.1111/1467-9663.00172

[5] : Gentrification.2013; 10.4324/9780203940877 10.4324/9780203940877

[6] : Introduction To The Forum: From Third To Fifth‐Wave Gentrification. *Tijdschrift voor Economische en Sociale Geografie* .2019;110(1) : 10.1111/tesg.12332 1-11 10.1111/tesg.12332

[7] : A gentrification stage‐model for London? Through the ‘lookingGlass ’ of Kensington. *Transactions of the Institute of British Geographers* .2025; 10.1111/tran.70012 10.1111/tran.70012

[8] : Rethinking Gentrification: Beyond the Uneven Development of Marxist Urban Theory. *Environment and Planning D: Society and Space* .1984;2(1) : 10.1068/d020047 47-74 10.1068/d020047

[9] : Stage Models of Gentrification. *Urban Affairs Quarterly* .1990;25(4) : 10.1177/004208169002500406 620-639 10.1177/004208169002500406

[10] : Gentrification in Latin America: addressing the politics and geographies of displacement. *Urban Geography* .2016;37(8) : 10.1080/02723638.2015.1103995 1175-1194 10.1080/02723638.2015.1103995

# Reviewer report: Reviewer response for version 1

**DOI:** 10.21956/openreseurope.22038.r55313

**Published:** 2025-06-27

**Authors:** Luis Lozano-Paredes

**Affiliations:** 1 Transdisciplinary School, University of Technology Sydney,, Sydney, NSW, Australia

**Summary**

 This essay introduces the concept of "platform gentrification" as a structural mutation in the production of urban inequalities under platform capitalism. The author reinterprets the classical four characteristics of gentrification through the lens of platform urbanism, examining how digital platforms mediate and restructure urban life. The paper makes a significant theoretical contribution by proposing platform gentrification not as a new typology but as a critical framework for understanding algorithmic mediation and data-driven infrastructures in urban transformation.

**Comments:**

**1. Theoretical Contribution**

 The concept of platform gentrification represents a significant theoretical advancement in understanding contemporary urban transformations. The author effectively bridges gentrification theory with platform urbanism literature, creating a framework that captures the unique characteristics of digitally mediated urban change. The reinterpretation of Davidson and Lees' (2005) four characteristics through a platform lens is particularly insightful, demonstrating how digital intermediation fundamentally alters traditional gentrification processes.

 However, the paper would benefit from a more explicit engagement with how platform gentrification differs from or relates to other recent conceptualisations by the author, such as "touristification". While the author mentions these briefly, a clearer delineation of the specific contributions of the platform gentrification framework would strengthen the theoretical argument.

**Empirical Grounding**

 While this is positioned as a theoretical and conceptual essay, the lack of empirical examples as case studies weakens some of the arguments. The author makes broad claims about platform impacts across "multiple global cities", including Lisbon, Barcelona, Mexico City, Rome, and New York, but provides limited concrete evidence. I would encourage the author to include more specific case studies, even if not empirical research from the author, that can illustrate how platform gentrification and, in general, the platform economy and urbanism manifest differently across contexts.

 For instance, labour around the global south on communities that actively resist and reshape platform logics through informal adaptations could be helpful. This suggests that platform gentrification may not be as unidirectional as the paper implies. Including such counterexamples would enrich the analysis.

**Global South Perspectives**

 The paper acknowledges platform gentrification as a "global phenomenon," but primarily draws on examples and literature from the Global North. This represents a significant limitation. Platform urbanism operates very differently in cities of the Global South, where informal economies, weak institutional frameworks, and community-driven adaptations create alternative pathways.

 I strongly recommend incorporating more perspectives from Latin America, Africa, and Asia, where platform adoption often involves creative appropriation rather than passive acceptance. This would help avoid reproducing the Global North bias that characterises much platform urbanism scholarship.

**The Four Characteristics Analysis**

 The reinterpretation of gentrification's four characteristics is well-executed but could be enhanced:

1. **Capital reinvestment:** The discussion of digital landlords and proptech is compelling. However, it overlooks how communities create alternative financial circuits through platform adaptations.
2. **New social groups:** The characterisation of platform users as possessing "strong digital capital" may not apply universally. In many Global South contexts, platform workers often lack digital capital but develop it through collective learning.
3. **Landscape transformation:** The concept of "aesthetic serialization" is fascinating but needs more empirical support. How exactly do we measure or observe this algorithmic aestheticization?
4. **Displacement:** The notion of "anticipatory displacement" is innovative and well-argued. This represents one of the paper's most substantial contributions.

**Minor Comments**

**Conceptual Clarity**

- The relationship between platform gentrification and platform capitalism needs more precise articulation. Are all platform capitalist interventions necessarily gentrifying?

**Methodological Considerations**

 While this is a theoretical essay, some discussion of how to empirically study platform gentrification would be valuable. What methods might researchers use to identify and analyse these processes?

**Writing and Structure**

 The essay is generally well-written, but could benefit from:

- Clearer transitions between sections
- More consistent use of examples throughout (not just in certain sections)
- A stronger conclusion that outlines specific research directions

**Recommendations**

1. **Expand geographical scope:** Include cases from the Global South to test the universal applicability of the framework.
2. **Address community agency:** Discuss how communities resist, adapt, or subvert platform gentrification processes.
3. **Develop methodological guidelines:** Provide suggestions for how researchers might empirically investigate platform gentrification, which could be a conceptual contribution to methodological innovation within the platform economy and platform studies.
4. **Clarify scope conditions:** Under what circumstances does platform gentrification occur? Are there cases where platforms don't lead to gentrification?
5. **Engage with platform cooperativism:** The essay would benefit from discussing alternative platform models that might mitigate gentrifying effects.

**Questions for the Author:**

1. *How does platform gentrification relate to broader processes of financialisation in cities?*
2. *Can you provide specific examples of "anticipatory displacement" in action?*
3. *How might regulatory frameworks address platform gentrification while preserving innovation benefits?*
4. *What role do platform workers themselves play in either facilitating or resisting gentrification?*
**Reviewer Questions:**
**Is the topic of the essay discussed accurately in the context of the current literature?**
**Partly**
  The author demonstrates a strong command of the literature on gentrification and platform urbanism, particularly scholarship from the Global North. The engagement with foundational works by authors like Davidson and Lees, Smith, and recent platform urbanism scholars is thorough and accurate. However, there is a significant gap in the literature review regarding Global South scholarship on platform urbanism and alternative platform models. The essay would benefit from incorporating works on informal platform adaptations, community-driven technologies, and southern perspectives on digital urban transformation. This geographical limitation affects the completeness of the literature contextualisation.
**Is the work clearly and cogently presented?**
**Yes**
  The essay follows a logical structure, moving from establishing the concept of platform gentrification to systematically reinterpreting the four classical characteristics of gentrification. The writing is generally clear, and the academic concepts are well-explained. The theoretical framework is presented in an accessible manner, making complex ideas understandable. Minor improvements could include clearer transitions between sections and more consistent use of examples throughout, but overall, the presentation is effective and coherent.
**Is the argument persuasive and supported by appropriate evidence?**
**Partly**
  The theoretical argument for platform gentrification as a new analytical framework is intellectually persuasive and well-constructed. The reinterpretation of gentrification's four characteristics through a platform lens offers compelling insights, particularly concepts like "anticipatory displacement" and "aesthetic serialization." However, the empirical support is uneven. While some claims are well-supported with references to specific platforms and their effects, other assertions about the impacts of platforms across multiple global cities lack concrete evidence. The essay would be more persuasive with additional empirical examples demonstrating how platform gentrification manifests in different urban contexts.
**Does the essay contribute to the cultural, historical, social understanding of the field?**
**Yes**
  Despite its limitations, this essay makes a significant contribution to understanding contemporary urban transformation. The concept of platform gentrification provides a valuable framework for analysing how digital intermediation is reshaping cities in ways that traditional gentrification theory cannot fully capture. The essay advances our understanding of how algorithmic governance, data-driven decision-making, and platform logic contribute to the creation of new forms of urban inequality and displacement. This theoretical contribution will be valuable for researchers studying the intersection of technology and urban change, even as it requires further empirical development and geographical expansion.
**Aspects Not Assessed**
  I was unable to fully assess the empirical validity of claims about specific cities mentioned (Lisbon, Barcelona, etc.), as limited evidence is provided.
**Overall Assessment**
  This essay makes a significant theoretical contribution to platform studies and platform urbanism literature. The concept of platform gentrification offers a valuable framework for understanding contemporary urban transformations. However, the paper would be significantly strengthened by incorporating Global South perspectives, providing more empirical grounding, and addressing community agency in platform processes.
  The author demonstrates a strong theoretical sophistication and a thorough knowledge of the relevant literature. With the suggested revisions, this work could become a foundational text in the field of platform studies.
**Approved with Reservations**
  The paper makes critical theoretical contributions but requires revisions to address geographical limitations, provide more empirical support, and incorporate perspectives on community agency and resistance. Once these issues are addressed, this will be a valuable addition to the platform urbanism literature.

Is the argument persuasive and supported by appropriate evidence?

Partly

Is the work clearly and cogently presented?

Yes

Is the topic of the essay discussed accurately in the context of the current literature?

Partly

Does the essay contribute to the cultural, historical, social understanding of the field?

Yes

Reviewer Expertise:

Design Research, Platform Studies, Science and Technology Studies (STS), Technology Governance, Urban Studies

I confirm that I have read this submission and believe that I have an appropriate level of expertise to confirm that it is of an acceptable scientific standard, however I have significant reservations, as outlined above.
